# Preconception Maternal and Paternal Exposure to Persistent Organic Pollutants and Birth Size: The LIFE Study

**DOI:** 10.1289/ehp.1308016

**Published:** 2014-08-05

**Authors:** Candace A. Robledo, Edwina Yeung, Pauline Mendola, Rajeshwari Sundaram, Jose Maisog, Anne M. Sweeney, Dana Boyd Barr, Germaine M. Buck Louis

**Affiliations:** 1 Division of Intramural Population Health Research, *Eunice Kennedy Shriver* National Institute of Child Health and Human Development, National Institutes of Health, Department of Health and Human Services, Rockville, Maryland, USA; 2 Department of Epidemiology and Biostatistics, School of Rural Public Health, Texas A&M Health Sciences Center, College Station, Texas, USA; 3 Rollins School of Public Health, Emory University, Atlanta, Georgia, USA

## Abstract

Background: Persistent organic pollutants (POPs) are developmental toxicants, but the impact of both maternal and paternal exposures on offspring birth size is largely unexplored.

Objective: We examined associations between maternal and paternal serum concentrations of 63 POPs, comprising five major classes of pollutants, with birth size measures.

Methods: Parental serum concentrations of 9 organochlorine pesticides, 1 polybrominated biphenyl (PBB), 7 perfluoroalkyl chemicals (PFCs), 10 polybrominated diphenyl ethers (PBDEs), and 36 polychlorinated biphenyls (PCBs) were measured before conception for 234 couples. Differences in birth weight, length, head circumference, and ponderal index were estimated using multiple linear regression per 1-SD increase in natural log-transformed (ln-transformed) chemicals. Models were estimated separately for each parent and adjusted for maternal age, maternal prepregnancy body mass index (kilograms per meter squared) and other confounders, and all models included an interaction term between infant sex and each chemical.

Results: Among girls (*n* = 117), birth weight was significantly lower (range, 84–195 g) in association with a 1-SD increase in ln-transformed maternal serum concentrations of DDT, PBDE congeners 28 and 183, and paternal serum concentrations of PBDE-183 and PCB-167. Among boys (*n* = 113), maternal (PCBs 138, 153, 167, 170, 195, and 209 and perfluorooctane sulfonamide) and paternal (PCBs 172 and 195) serum concentrations of several POPs were statistically associated with lower birth weight (range, 98–170 g), whereas paternal concentrations of PBDEs (66, 99) were associated with higher birth weight. Differences in offspring head circumference, length, and ponderal index were also associated with parental exposures.

Conclusions: Preconceptional maternal and paternal concentrations of several POPs were associated with statistically significant differences in birth size among offspring.

Citation: Robledo CA, Yeung E, Mendola P, Sundaram R, Maisog J, Sweeney AM, Barr DB, Buck Louis GM. 2015. Preconception maternal and paternal exposure to persistent organic pollutants and birth size: the LIFE Study. Environ Health Perspect 123:88–94; http://dx.doi.org/10.1289/ehp.1308016

## Introduction

The presence of persistent organic pollutants (POPs) in maternal blood (Llop et al. 2010; Rodríguez-Dozal et al. 2012; Rudge et al. 2012; Wang et al. 2009), umbilical cord blood (Arbuckle et al. 2013; Foster et al. 2011), and breast milk (Mikeš et al. 2012; Pan et al. 2009; Tanabe and Kunisue 2007) documenting *in utero* and lactational exposure has prompted epidemiological studies to examine the relationship between exposure to these compounds and fetal growth and development (Mattison 2010; Windham and Fenster 2008). Research in this area has generally focused on outcomes such as birth weight and length of gestation, strong indicators of neonatal health. Epidemiological studies have shown a decrease in birth weight in relation to exposure to POPs that include polychlorinated biphenyls (PCBs) (Govarts et al. 2012; Karmaus and Zhu 2004; Murphy et al. 2010), polychlorinated diphenyl ethers (PBDEs) (Harley et al. 2011), perfluoralkyl chemicals (PFCs) (Washino et al. 2009), and organochlorine pesticides (OCPs) (Wolff et al. 2007). Although the previous studies have demonstrated an association between POPs and reduced birth size, proxied by birth weight, findings are inconsistent and studies have also reported null associations (Farhang et al. 2005; Givens et al. 2007; Karmaus and Zhu 2004; Kezios et al. 2012; Longnecker et al. 2005; Mazdai et al. 2003; Olsen et al. 2009; Pan et al. 2009; Sweeney and Symanski 2007; Tan et al. 2009; Wu et al. 2010).

Inconsistences may be attributed to several key limitations of prior studies. Past research has focused on pre- and postnatal exposures to POPs, despite evidence that the preconception period may be a critical window of exposure for fetal growth and development (Chapin et al. 2004). Given the metabolic and physiological changes that occur during pregnancy, preconception levels may be more accurate in capturing the dose to the fetus. Regardless of their long half-lives and persistent nature, the concentrations of pollutants may vary across critical windows of development, as seen with PCBs (Bloom et al. 2007) and other selected POPS (Wang et al. 2009). Finally, prior studies have focused on elucidating the impact of maternal exposure to POPs on birth size, regardless of the fact that pregnancy is a couple-dependent outcome. Consequently, the impacts of paternally mediated factors on birth size have been largely unstudied (Cordier 2008; Shah and Knowledge Synthesis Group on Determinants of Preterm/Low Birthweight Births 2010). Limited to occupational studies, little is known about the impact of paternal POP exposures on birth size (Lawson et al. 2004; Michalek et al. 1998).

We aim to address these gaps in knowledge by estimating the associations of maternal and paternal preconceptional serum concentrations of POPs on birth size. We hypothesize that preconceptional serum concentrations of both maternal and paternal persistent environmental chemicals are associated with reduced birth size measures (i.e., birth weight, head circumference, length, and ponderal index).

## Methods

*Study population*. The Longitudinal Investigation of Fertility and the Environment (LIFE) Study was a prospective cohort study conducted between 2005 and 2009 to assess the impact of persistent environmental chemicals on reproductive outcomes (Buck Louis et al. 2011). Briefly, LIFE recruited couples (*n* = 501) who resided in Michigan and Texas with reported or presumed exposure to persistent environmental chemicals. Married couples or those in a committed relationship who were planning a pregnancy in the subsequent 6 months were targeted for recruitment. Couples were ineligible to participate if either partner was medically/surgically sterile; they had discontinued contraception for > 2 months; the female’s menstrual cycle was not between 21 and 42 days or she had received injectable contraceptives within the previous 12 months; they were not of reproductive age (females < 18 or > 40 years, and males < 18 years); or they could not communicate in English or Spanish. Couples were followed until a positive human chorionic gonadotropin (hCG) pregnancy test or through 12 months of attempting pregnancy. Following conception, women were followed daily for 8 weeks and then monthly until a pregnancy loss or delivery. Analyses were restricted to couples for whom a singleton delivery was observed (*n* = 247), regardless of a previous loss, and for whose child birth weight was reported (*n* = 234). In doing so, we excluded data for two sets of twin births.

Institutional review board approval was obtained from all collaborating institutions, and informed written consent was obtained from all couples before their participation.

*Assessment of fetal growth outcomes and covariates*. Couples were asked to report birth size characteristics for the index birth using standardized birth announcements specifically designed for the LIFE study that were included in the pregnancy diary (available on request). Women were trained in their use and completed them after delivery. Information recorded on the delivery cards included infant sex, birth weight (in grams or pounds and ounces) (*n* = 230), length (in centimeters or inches) (*n* = 229), and head circumference (in centimeters or inches) (*n* = 181). Ponderal index (*n* = 229), a marker of asymmetrical growth retardation thought to be a result of fetal insult, was defined as 100 × [birth weight (grams)/length (cubic centimeters)] (Sparks et al. 1998). Analyses did not include infants whose birth weight (*n* = 2) or head circumference (*n* = 2) exceeded the 99th percentile.

Baseline questionnaires were administered to each partner separately and were used to collect medical and reproductive histories. Information on lifestyle factors such as the use of alcohol and tobacco in the previous 12 months was also collected. Participants then underwent an anthropometric assessment (Lohman et al. 1988) for measurement of their height and weight. Body mass index (BMI) was calculated as weight (kilograms) divided by height (meters squared).

Daily journals captured data on lifestyle factors, sexual intercourse, and home pregnancy test results. A fertility monitor captured data on peak luteinizing hormone concentrations indicative of ovulation. These data combined with information on sexual intercourse and positive hCG pregnancy test results allowed for the estimation of day of conception and, thereafter, gestational age. Pregnant women were asked to complete daily pregnancy journals that captured information on weight gain and gravid diseases (e.g., gestational diabetes).

*Exposure assessment*. Biospecimens were collected from each partner during the baseline home visit. Approximately 20 mL of nonfasting blood were collected to measure concentrations of environmental chemicals. For quality control, blood collection equipment was tested and determined to be free from contaminants under study. Quantification of serum toxicants was conducted by the Division of Laboratory Sciences in the National Center for Environmental Health at the Centers for Disease Control and Prevention (CDC). A list of all congeners measured and their abbreviations can be found in Table 1. Established protocols using isotope dilution gas chromatography–high resolution mass spectrometry or high performance liquid chromatography–tandem mass spectrometry (Barr et al. 2003; Kuklenyik et al. 2005; Sandau et al. 2003) were used to estimate serum concentrations of 1 polybrominated biphenyl (PBB), 9 OCPs, 10 PBDEs, 36 PCBs, and 7 PFCs. Liquid chromatography–isotope dilution tandem mass spectrometry (Bernert et al. 1997) was used to quantify serum concentrations of cotinine (nanograms per milliliter), as a measure of tobacco exposure. Enzymatic methods were used to estimate total cholesterol, nonesterified cholesterol, triglycerides, and phospholipids (Akins et al. 1989). Total serum lipids (nanograms per gram serum) were calculated using established summation methods (Bernert et al. 2007; Phillips et al. 1989). Serum lipid concentrations were included in models as a covariate, and pollutant concentrations are reported in nanograms per gram serum, except for PFCs and cotinine, which are reported in nanograms per milliliter.

**Table 1:** Persistent organic pollutants (POPs) measured in study population, LIFE Study, 2005–2008.

POPs	Compounds or congeners
Polybrominated biphnenyl (PBB)	PBB-153
Organochlorine pesticides (OCPs)	Hexachlorobenzene (HCB), β-hexachlorocyclohexane (β-HCH), γ-hexachlorocyclohexane (γ-HCH), oxychlorodane, *trans*-nonachlor, mirex, *o,p*´-DDT, *p,p*´-DDT, and *p,p*´-DDE
Polybrominated diphenyl ethers (PBDEs)	PBDEs 17, 28, 47, 66, 85, 99, 100, 153, 154, 183
Polychlorinated biphenyls (PCBs)	PCBs 28, 44, 49, 52, 66, 74, 87, 99, 101, 105, 110, 114, 118, 128, 138, 146, 149, 151, 153, 156, 157, 167, 170, 172, 177, 178, 180, 183, 187, 189, 194, 195, 196, 201, 206, 209
Perfluoroalkyl chemicals (PFCs)	2-(*n*-ethyl-perfluorooctane sulfonamide) acetate (Et-PFOSA-AcOH), 2-(*N*-methyl-perfluorooctane sulfonamide) acetate (Me-PFOSA-AcOH), perfluorodecanoate (PFDeA), perfluorononanoate (PFNA), perfluorooctane sulfonamide (PFOSA), perfluorooctane sulfonate (PFOS), and perfluorooctanoate (PFOA)

*Statistical analyses*. We assessed the distributions of all exposures and relevant covariates. Normality of continuous variables was assessed using Kolmogorov–Smirnov tests. Missing covariate values and missing chemical, cotinine, and lipid data (< 4%), due to insufficient blood for analysis, were imputed under the missing at random assumption, using Markov chain Monte Carlo methods (Rubin 1996) detailed elsewhere (Buck Louis et al. 2013). Machine-read values for chemical concentrations were used, and values below the limit of detection were not substituted to avoid introducing bias (Schisterman et al. 2006). To account for skewed distribution and for ease of interpretation, chemical concentrations were natural log-transformed (ln) and rescaled by their standard deviation. Geometric means (GMs) and 95% confidence intervals (CIs) were calculated for all chemicals. See Supplemental Material, Table S1, for a list of SDs and GMs for this study population. The outcome variables, birth weight (grams), head circumference (centimeters), length (centimeters), and ponderal index (grams per cubic centimeter) were not ln-transformed. Each outcome and chemical concentration was modeled as a continuous variable.

We used multiple linear regression to estimate the mean difference in each outcome per 1-SD increase for all ln-transformed chemicals. The mean differences in growth outcomes for each chemical and parent were estimated separately. Models were adjusted *a priori* for maternal age, the difference between maternal and paternal age, maternal prepregnancy BMI, infant sex, serum lipids (except PFCs), and serum cotinine concentrations (Cliver et al. 1995; Cogswell and Yip 1995; Shah and Knowledge Synthesis Group on Determinants of Preterm/Low Birthweight Births 2010). The sum of the remaining chemical concentrations (ln-transformed and scaled by their respective standard deviation) in each chemical’s respective class was included in models to account for the mean level of individual concentrations. To account for the partner’s exposure, each model also included the total sum of partner’s serum concentrations in the respective class for the chemical being evaluated. Interaction between each pollutant and infant sex was evaluated by examining the statistical significance of their product term in each model (*p* < 0.05). Evidence of interaction between chemical exposures and birth outcomes by infant sex was observed for some chemicals and models by significant interaction term (*p*-interaction < 0.05), so all associations were estimated stratified by sex for consistency. We report associations for chemicals for which at least one statistically significant association was estimated with birth size measures. Statistical significance was set at *p* < 0.05.

We conducted a sensitivity analysis that excluded pregnancies complicated by gestational diabetes or hypertension because of their known effects on fetal growth (Mayer and Joseph 2013). Results and conclusions of the association between parental preconception exposure to persistent organic pollutants and birth size measures did not vary (data not shown). Therefore, model estimates that include all pregnancies are reported.

## Results

*Study population*. Partners for whom a singleton delivery occurred were very similar in their sociodemographic characteristics (Table 2). The majority of men and women were non-Hispanic white, had a college education, were insured, and did not smoke or drink alcohol. Compared with their female counterparts, males were approximately 2 years older and on average had a higher BMI (29.3 vs. 26.5 kg/m^2^). Women reported having gestational diabetes mellitus (*n* = 27), hypercholesterolemia (*n* = 18), and preexisting hypertension (*n* = 7). The majority of infants were girls (51%). The mean (± SD) postconception gestational age and birth weight of infants at delivery was 36.2 ± 2.2 weeks and 3382.3 ± 487.5 g, respectively.

**Table 2:** Description of study cohort by partner among those with a singleton delivery (*n* = 234), LIFE Study, 2005–2009 [*n* (%) or mean ± SD].

Characteristic	Mother	Father
Race/ethnicity
Non-Hispanic white	194 (84)	198 (85)
Non-Hispanic black	2 (1)	4 (2)
Hispanic	20 (9)	20 (9)
Other	16 (7)	12 (5)
Education
< High school	0 (0)	2 (1)
High school/equivalent	9 (4)	5 (2)
College	223 (96)	225 (97)
Health insurance
No	5 (2)	10 (4)
Yes	227 (98)	223 (96)
Smoking status at baseline*
Active (cotinine ≥ 100 ng/mL)	11 (5)	24 (10)
Passive (cotinine < 100 ng/mL)	219 (95)	205 (90)
Cigarettes smoked (9–12 weeks)
None	227 (98.8)	—
< 10	2 (0.8)	—
≥ 10	1 (0.4)	—
Alcohol use at baseline^*a*^^,^*
No	52 (22)	31 (13)
Yes	182 (78)	203 (87)
Alcohol use (9–12 weeks)
None	229 (99.6)	—
1 drink/week	1 (0.4)	—
Age (years)*	29.8 ± 3.7	31.5 ± 4.6
Body mass index (kg/m^2^)*	26.4 ± 6.5	29.3 ± 5.3
Gravidity	1.1 ± 1.2	1.0 ± 1.1
Parity	0.7 ± 0.8	0.69 ± 0.8
All characteristics are self-reported except for body mass index. Missing covariate data was not included in table. ^***a***^At baseline, participants reported whether they had consumed alcohol in the last 12 months. *Chi-square test *p* < 0.05.		

Serum concentrations of most POPs among couples were similar for partners. However, geometric mean concentrations of pesticides such as *p,p*´-DDE (dichlorodiphenyldichloroethylene) (0.580 ng/g; 95% CI: 0.534, 0.630 vs. 0.752 ng/g; 95% CI: 0.700, 0.808), mirex (0.007 ng/g; 95% CI: 0.007, 0.008 vs. 0.013 ng/g; 95% CI: 0.011, 0.014), and several PFCs were markedly higher among males (see Supplemental Material, Table S1). Preconceptional parental concentrations of POPs were found to be associated with changes in birth size measures.

*OCPs and PBB*. Statistically significant differences in birth size measures were estimated in association with both maternal and paternal preconception serum concentrations of OCPs among girls, but virtually no significant associations were observed among boys (Table 3). See Supplemental Material, Tables S2–S5, for all estimated associations for birth size measures and chemicals evaluated in our study. Among girls, a 1-SD increase in ln-transformed maternal serum concentrations of *o,p*´-DDT (dichlorodiphenyltrichloroethane) was associated with lower birth weight (β = 195.39 g; 95% CI: –351.25, –39.52), driven perhaps by smaller head circumference (β = –0.78 cm; 95% CI: –1.48, –0.09). Smaller head circumference was also seen with increasing maternal concentrations of β-HCH (hexachlorocyclohexane) (β = 1.47 cm; 95% CI: –2.33, –0.61). Length among girls was inversely associated with maternal concentrations of γ-HCH (lindane) and subsequently higher ponderal index (β = 0.09 g/cm^3^; 95% CI: 0.03, 0.16). Similarly, paternal concentrations of γ-HCH were associated with shorter length and higher ponderal index among girls, despite mutual adjustment for mean partner concentrations of other organochlorine exposure. A higher ponderal index among girls was also seen with increasing paternal concentrations of *p,p*´-DDE. Except for larger head circumference observed with increasing maternal concentrations of HCB (hexachlorobenzene), preconceptional parental concentrations of OCPs were not associated with birth size among boys. Parental preconceptional concentrations of PBB-153 were not found to be associated with birth size measures.

**Table 3:** Adjusted^*a*^ mean changes (β) and their 95% CIs for each birth size measure per 1-SD increase in ln-transformed chemical concentration^*b*^ by partner and infant sex, LIFE Study, 2005–2009.

Outcome	Maternal		aternal	
	Girl β (95% CI)	Boy β (95% CI)	Girl β (95% CI)	Boy β (95% CI)
Birth weight (g)^*c*^
*o,p*´-DDT	–195.39 (–351.25, –39.52)*	–6.11 (–93.02, 80.80)*	–49.28 (–153.83, 55.27)	4.32 (–86.15, 94.79)
PBDE-28	–151.33 (–298.56, –4.10)	–64.65 (–164.92, 35.63)	–30.85 (–154.83, 93.14)	14.99 (–99.94, 129.93)
PBDE-66	–21.98 (–141.29, 97.33)*	125.04 (18.16, 231.92)*	–39.80 (–152.57, 72.96)	–47.85 (–173.89, 78.19)
PBDE-99	52.08 (–120.80, 224.96)	133.39 (9.12, 257.37)	17.53 (–123.74, 158.80)	59.43 (–90.47, 209.33)
PBDE-183	–84.60 (–154.39, –14.82)*	85.21 (–32.32, 202.74)*	–92.13 (–173.44, –10.82)	21.32 (–85.27, 127.91)
PCB-138	–82.30 (–219.22, 54.61)	–149.6 (–285.16, –14.06)	–69.04 (–191.78, 53.71)	–103.02 (–264.04, 57.99)
PCB-153	–90.94 (–240.89, 59.01)	–169.93 (–317.32, –22.53)	–29.33 (–164.37, 105.70)	–68.77 (–226.45, 88.91)
PCB-167	–61.69 (–172.52, 49.15)	–129.24 (–228.16, –30.31)	–97.49 (–187.45, –7.54)	–38.24 (–139.86, 63.37)
PCB-170	–80.87 (–223.93, 62.18)	–153.69 (–288.45, –18.92)	–16.85 (–144.38, 110.67)	–119.29 (–268.37, 29.79)
PCB-172	68.59 (–48.81, 185.99)	–37.21 (–148.16, 73.74)	–35.87 (–143.12, 71.38)	–166.89 (–311.19, –22.60)
PCB-195	–18.46 (–128.10, 91.18)	–137.73 (–259.57, –15.89)	–6.94 (–102.96, 89.07)*	–148.39 (281.69, –15.08)*
PCB-209	–24.96 (–135.66, 85.74)	–98.88 (–187.14, –10.61)	–24.49 (–124.53, 75.56)	–0.80 (–115.85, 114.24)
PFOSA	–8.80 (–93.55, 75.95)	–104.23 (–194.16, –14.30)	10.48 (–85.29, 106.26)	–73.76 (–154.43, 6.91)
Length (cm)^*d*^
γ-HCH	–0.59 (–1.14, –0.03)*	0.34 (–0.17, 0.84)*	–0.51 (0.98, –0.04)*	0.33 (–0.20, 0.86)*
PBDE-28	–1.14 (–2.00, –0.28)*	–0.18 (–0.76, 0.41)*	–0.28 (–1.02, 0.46)	0.00 (–0.67, 0.67)
PBDE-99	0.25 (–0.76, 1.27)	0.76 (0.04, 1.48)	0.18 (–0.65, 1.01)	0.44 (–0.43, 1.31)
PCB-167	–0.47 (–1.12, 0.19)	–0.42 (–1.00, 0.16)	–0.57 (–1.12, –0.02)	–0.11 (–0.70, 0.49)
Head circumference (cm)^*e*^
HCB	0.10 (–0.40, 0.61)	0.44 (0.01, 0.87)	0.12 (–0.40, 0.63)	–0.22 (–0.63, 0.19)
β-HCH	–1.47 (–2.33, –0.61)*	–0.22 (–0.58, 0.14)*	0.23 (–0.68, 1.13)	–0.16 (–0.78, 0.46)
*o,p*´-DDT	–0.78 (–1.48, –0.09)	–0.06 (–0.47, 0.35)	–0.14 (–0.59, 0.32)	0.20 (–0.28, 0.67)
PBDE-28	–1.05 (–1.73, –0.38)*	–0.24 (–0.67, 0.19)*	0.02 (–0.57, 0.61)	0.18 (–0.33, 0.69)
PBDE-66	–0.17 (–0.76, 0.41)*	0.60 (0.02, 1.18)*	0.10 (–0.39, 0.59)	0.33 (–0.26, 0.92)
PBDE-85	0.34 (–0.78, 1.45)*	1.04 (0.04, 2.03)*	–0.01 (–0.83, 0.80)	0.16 (–0.76, 1.09)
PBDE-99	0.27 (–0.48, 1.02)	0.91 (0.23, 1.60)	0.10 (–0.52, 0.71)	0.31 (–0.38, 0.99)
PCB-128	0.08 (–0.30, 0.45)*	–0.86 (–1.45, –0.10)*	–0.18 (–0.61, 0.25)	–0.66 (–1.31, –0.01)
PCB-138	–0.65 (–1.25, –0.05)	–0.67 (–1.67, –0.06)	–0.32 (–0.86, 0.22)	–0.56 (–1.30, 0.17)
PCB-153	–0.65 (–1.30, 0.01)	–0.78 (–1.27, –0.08)	0.06 (–0.59, 0.71)	–0.22 (–0.79, 0.36)
PCB-157	–0.22 (–0.62, 0.18)	–0.17 (–0.66, 0.32)	–0.06 (–0.46, 0.34)	–0.54 (–1.01, –0.06)
PCB-167	–0.04 (–0.55, 0.46)	–0.47 (–0.95, 0.00)	–0.45 (–0.86, –0.03)	–0.32 (–0.80, 0.16)
PCB-201	0.51 (0.08, 0.93)	0.28 (–0.31, 0.87)	0.19 (–0.22, 0.61)	–0.32 (–1.08, 0.44)
PCB-206	0.52 (0.06, 0.98)	0.16 (–0.30, 0.63)	0.16 (–0.34, 0.66)	–0.38 (–1.17, 0.41)
Ponderal Index (g/cm^3^)^*d*^
γ-HCH	0.09 (0.03, 0.16)*	–0.01 (–0.07, 0.05)*	0.08 (0.02, 0.13)	0.00 (–0.07, 0.06)
*p,p*´-DDE	0.03 (–0.07, 0.13)	0.03 (–0.04, 0.09)	0.12 (0.02, 0.22)*	0.01 (–0.06, 0.07)*
PCB-138	–0.10 (–0.20, –0.01)	–0.13 (–0.23, –0.04)	–0.09 (–0.18, 0.00)	–0.13 (–0.24, –0.02)
PCB-156	–0.04 (–0.11, 0.03)	–0.05 (–0.13, 0.03)	–0.08 (–0.16, –0.01)	–0.11 (–0.20, –0.03)
PCB-157	–0.03 (–0.09, 0.04)	–0.04 (–0.12, 0.03)	–0.03 (–0.09, 0.04)	–0.08 (–0.16, –0.01)
PCB-170	–0.10 (–0.20, 0.00)	–0.10 (–0.20, –0.01)	–0.02 (–0.11, 0.07)	–0.03 (–0.14, 0.07)
PCB-172	–0.05 (–0.13, 0.03)	–0.09 (–0.17, –0.02)	–0.01 (–0.09, 0.07)	–0.03 (–0.13, 0.07)
Et-PFOSA-AcOH	–0.09 (–0.16, –0.02)	–0.02 (–0.08, 0.04)	–0.04 (–0.10, 0.03)	–0.07 (–0.16, 0.03)
^***a***^Results are presented only for chemicals for which at least one statistically significant association between pollutants and birth size measures were estimated. Models are adjusted for maternal and paternal serum lipids, serum cotinine, maternal prepregnancy BMI (kg/m^2^), maternal age, difference in parental age, infant sex, and the individual and partner sum of remaining chemical concentrations in each chemical’s respective class. ^***b***^Restricted to chemicals with a significant association with fetal growth outcomes. ^***c***^Data for 113 boys and 117 girls were available for analysis. ^***d***^Data for 113 boys and 116 girls were available for analysis. ^***e***^Data for 90 boys and 91 girls were available for analysis. *p < 0.05 for parent concentration and infant sex interaction term.				

*PFCs*. The mean birth weight of boys was 104.23 g lower (95% CI: –194.16, –14.30) for every 1-SD increase in ln-transformed maternal concentrations of PFOSA (perfluorooctane sulfonamide). Maternal concentrations of the Et-PFOSA-AcOH [2-(*n*-ethyl-perfluorooctane sulfonamide) acetate] metabolite were associated with a smaller mean ponderal index among girls (–0.09 g/cm^3^; 95% CI: –0.16, –0.02). We did not observe associations between preconceptional paternal concentrations of PFCs and birth measures. Furthermore, preconceptional parental concentrations of PFCs were not associated with length or head circumference at birth.

*PBDEs*. Maternal concentrations of PBDEs were associated with significant differences in mean birth weight in boys and girls. Maternal concentrations of PBDE congeners 28 and 183 were associated with lower birth weight among girls; the largest negative association was estimated for PBDE-28 (β = –151.33 g; 95% CI: –298.56, –4.10). Maternal concentrations of PBDE-28 were also statistically associated with smaller length and head circumference among girls. On the contrary, for every 1-SD increase in ln-transformed maternal concentrations of PBDEs 66 and 99, mean birth weight among boys was 125.04 g (95% CI: 18.16, 231.92) and 133.39 g (95% CI: 9.12, 257.37) higher, respectively. Among boys, PBDE congeners were also statistically associated with larger length (PBDE-99) and head circumference (PBDEs 66, 85, 99). As seen with maternal concentrations, paternal concentrations of PBDE-183 were also significantly associated with lower birth weight among girls (β = –92.13 g; 95% CI: –173.44, –10.82).

*PCBs*. Among girls, maternal concentrations of PCBs were not associated with significant differences in birth weight. However, for every 1-SD increase in ln-transformed concentrations of paternal concentrations of PCB-167, the mean birth weight among girls was 97.49 g lower (95% CI: –187.45, –7.54), and mean length (β = –0.57 cm; 95% CI: –1.12, –0.02) and head circumference (β = –0.45 cm; 95% CI: –0.86, –0.03) were smaller. Significant associations between parental concentrations of PCBs and birth size were more frequent among boys. Birth weight among boys was lower by 99–170 g per 1-SD increase in ln-transformed maternal (PCBs 138, 153, 167, 170, 195, and 209) and paternal (PCBs 172, 195) concentrations. Maternal concentrations of PCBs were statistically associated with smaller head circumference in girls (PCB-138) and boys (PCBs 128, 138, 153). Paternal concentrations of PCBs were also significantly associated with smaller head circumference among girls (PCB-167) and boys (PCBs 128, 157). Maternal concentrations of PCBs 201 and 206 were associated with larger head circumference. Maternal concentrations of PCB-138 were associated with lower mean birth weight among boys and smaller mean head circumference and ponderal index among boys and girls. Paternal concentrations of PCB-138 were estimated to be associated with smaller mean ponderal index among boys. Additionally, in girls, paternal concentrations of PCB-156 and in boys maternal (PCBs 170, 172) and paternal (PCBs 156, 157) PCB concentrations were associated with smaller ponderal index (range, 0.08–0.13 g/cm^3^).

*Persistent organic pollutants associated with multiple birth size outcomes*. Both maternal and paternal concentrations of several persistent organic pollutants were associated with statistical differences in the same birth size measure among their offspring. The statistical differences associated with increasing parental concentrations of these pollutants were often of similar magnitude and direction. We briefly highlight these compounds here.

Lower mean birth weight was observed in association with increasing preconception maternal and paternal concentrations of PBDE-183 among girls and PCBs 128 and 195 among boys. Maternal concentrations of PCB-167 were associated with lower mean birth weight among girls only, but paternal concentrations were associated with lower birth weight in boys. Increasing maternal and paternal concentrations of γ-HCH were associated with smaller head circumference and higher ponderal index among girls.

## Discussion

In this prospective pregnancy study with preconception enrollment of couples, we demonstrated that both preconception maternal and paternal serum concentrations of persistent organic pollutants were significantly associated with birth size measures among their offspring, even after taking into account their partner’s serum concentrations. In addition, we also report several statistically significant differences in birth size measures by infant sex and between and within classes of pollutants. We observed decreases in infant birth weight between 85 and 195 g with 1-SD increases in preconception maternal and paternal serum concentrations of POPs.

This reduction is similar in magnitude to what has been reported for other prenatal maternal environmental exposures. Compared with nonsmokers, lower mean birth weight has been reported for infants born to women who reported cigarette smoking during the first trimester or throughout pregnancy (range, 55–189 g) (Cliver et al. 1995). Meta-analyses have reported lower birth weight among infants born to nonsmoking women exposed to environmental tobacco smoke (33 g; 95% CI: 16, 51) (Leonardi-Bee et al. 2011) and in association with increasing cord serum concentrations of PCB-153 (150 g; 95% CI: 50, 250) (Govarts et al. 2012). Last, a meta-analysis reported that when compared with lower exposure groups, women exposed to higher mean levels of indoor air pollution from solid fuel use had infants whose birth weight was approximately 96.6 g lower (95% CI: 68.5, 124.7) (Pope et al. 2010).

Our findings underscore the importance of designing epidemiological studies that ascertain preconception parental exposures in relation to birth size measures. In addition, given that paternal environmental exposures are often overlooked when examining the associations between parental exposures and fetal growth, there is a need for more comprehensive investigations of the associations between preconception paternal exposures and fetal growth and development. Both maternal and paternal serum concentrations of several pollutants (PBDE-183, PCBs 128, 138, 167, and 195, and γ-HCH) were associated with birth size measures, but more research is needed to investigate whether associations that were specific to paternal serum concentrations are relevant and can be confirmed in other populations.

Few prospective pregnancy studies report parental preconception serum concentrations of POPs, making it difficult to further evaluate our findings. The only known study to examine the association between preconception maternal PCB levels and birth weight was conducted using data obtained from a prospective cohort of New York women and their partners planning a pregnancy within the next 6 months (Murphy et al. 2010). After adjustment for maternal height, smoking, and infant sex, the birth weight of infants (*n* = 50) born to mothers with the highest concentrations of antiestrogenic PCBs [interquartile range (IQR): 0.23–0.33 ng/g serum] was approximately 471 g (95% CI: –890.2, –51.3) lighter than infants born to mothers with the lowest concentrations (IQR: 0.13–0.15 ng/g serum). This study also examined the association between infant birth weight and maternal antiestrogenic PCB concentrations from serum measured during the prenatal period (median, 6 weeks gestation). The mean difference in infant birth weight between women with the highest (IQR: 0.15–0.21 ng/g serum) and lowest (IQR: 0.07–0.09 ng/g serum) prenatal concentrations of maternal antiestrogenic PCBs was approximately 260 g less (β = –260.5; 95% CI: –667.4, 146.5) than what was reported for preconception levels (Murphy et al. 2010).

Given the debate about classifying chemicals by their action, which may also be a function of dose, we decided to examine each individually. By doing so, we did not make any assumptions regarding their hypothesized biologic activity or how compounds may interact with each other in mixture form. However, in our present study we report statistically significant associations between birth size measures and two PCB congeners. For one previously shown to be estrogenic (PCB-153), we found that maternal concentrations were significantly associated with lower birth weight and head circumference in boys; for another, shown to be antiestrogenic (PCB-156), we found paternal concentrations to be associated with lower ponderal index in both boys and girls (Cooke et al. 2001). We also observed associations between birth weight and lower serum concentrations of PCBs than what has been previously published in a study of New York anglers and their partners planning a pregnancy, mentioned above (Murphy et al. 2010). It has been shown that serum concentrations of POPs in the LIFE study population (Buck Louis et al. 2013) are lower than reported for the U.S. population (CDC 2014). This difference is not surprising given that concentrations of persistent chemicals increase with age and the LIFE cohort is comprised of couples of reproductive age, unlike the NHANES population that comprises women 12–85 years of age.

Our study also reports several positive associations between pollutants and birth size measures. Maternal concentrations of PBDEs 66 and 99 were associated with increased mean birth weight, length, and head circumference among boys only. Maternal concentrations of PCBs 201 and 206 and maternal and paternal concentrations of OCPs were associated with increased mean head circumference and ponderal index among girls only. Although they are not comparable to our study, other studies have reported positive associations between maternal prenatal levels of environmental chemicals. Maternal prenatal concentrations of total PCBs and PBBs (congener specific information not available) have been associated with higher birth weight (Sweeney and Symanski 2007). Positive associations between head circumference and length have also been reported for maternal prenatal levels of organophophate pesticides not evaluated in this study (Eskenazi et al. 2004). Associations reported by these studies also differed by sex. We are unable to explain these findings, but posit that they may reflect differing structural activity or biological activity of individual congeners, particularly given that associations differed by infant sex. Also, the windows of vulnerability for a fetus’s growth and development may differ by congener. We also speculate that these positive associations may be confounded by healthy behaviors such as the consumption of fish or antioxidant-rich foods. These healthy behaviors, although potential sources of parental POP exposure, may also positively influence fetal growth and development.

Our study addressed several key limitations of prior studies with equivocal findings of the association between prenatal exposure to POPs and birth size. For one, many studies ascertained prenatal exposure to POPs during late pregnancy using maternal serum concentrations at the time of delivery or using umbilical cord serum concentrations. These studies may not be capturing exposure during relevant windows of fetal growth and development. Prospective pregnancy cohort studies that recruit couples discontinuing contraception to become pregnant are rare, and this is the only way to examine the association between preconceptional exposures to POPs and human birth size. Despite their long half-lives, maternal serum concentrations of PCBs and selected POPs can vary across critical windows of human reproduction and development during pregnancy (Bloom et al. 2007; Wang et al. 2009). Preconceptional maternal serum concentrations are not influenced by the expansion of blood volume and changes in metabolism associated with normal pregnancy. Thus, we can explore associations with birth size measures in relation to exposure that reflects preconception and early pregnancy, a key window for these effects.

Prior studies have focused on maternal exposures and how they impact developmental health. Paternal exposures to POPs have been largely unstudied, and little is known about their potential impact on fetal development and growth. Environmental chemical exposures that occur during spermatogenesis may affect the quality of a father’s gametes, and therefore may affect the susceptibility and health of his offspring *in utero* or after birth (Olshan and Faustman 1993). Epigenetic changes in gametes caused by transient paternal exposures may be stably transferred to multiple generations (Anderson 2005; Fullston et al. 2013). Many studies have reported global epigenetic changes in different cell types in association with environmental chemical exposures (Curley et al. 2011). In regard to past research, few studies have addressed the burden faced by participants in collecting biological specimens, as well as the cost of analyzing samples. Therefore, studies of the association between environmental toxicants and birth size have focused on select chemicals or a specific class of compounds. In our study we were not limited to studying a particular class or select group of analytes. We were able to examine the association between birth measures of interest and 63 chemicals, comprising five major classes of POPs (OCPs, PBB, PBDEs, PCBs, and PFCs).

Because of sample size limitations, we were unable to examine the associations between parental preconception levels of POPs and growth restriction or low birth weight. However, we were still able to estimate statistically significant associations in this preconception cohort. Another limitation is the reliance on maternal reported birth measures, which may be biased. Although maternal recall of birth weight has been shown to be accurate and reliable (Adegboye and Heitmann 2008; Bat-Erdene et al. 2013; Buka et al. 2004), the normal flexed condition and head molding of the neonate at delivery may lead to measurement error of length (Shinwell and Shlomo 2003) and head circumference, respectively. However, measurement/reporting errors should not be correlated to chemical concentrations, and our estimates may have been biased toward the null. We also did not control for multiple comparisons, given the exploratory nature of this work and our intent to identify signals for the preconception window that would require follow-up research. For each outcome, 189 comparisons were undertaken. We would have expected to observe 10 significant findings by chance alone when assessing maternal and paternal exposures: We observed 16 for birth weight, 5 for length, 16 for head circumference, and 12 for ponderal index. We observed more than the expected number of significant results by chance alone, though we still urge caution in the interpretation of our findings.

To our knowledge, this study is the first to investigate preconceptional and paternal measures of environmental chemicals in association with birth size. Our findings suggest that many of these chemicals are associated with reduced birth size measures even at low levels of exposure, and support the need for continued rigor in reducing and perhaps eliminating exposures.

## Supplemental Material

(333 KB) PDF
