# Supplementary material for: Preconception Maternal and Paternal Exposure to Persistent Organic Pollutants and Birth Size: The LIFE Study
Source: Environ Health Perspect. 2014 Aug 5;123(1):88–94. doi: 10.1289/ehp.1308016 (PMC4286275; doi:10.1289/ehp.1308016)
Supplement: (333 KB) PDF [file ehp.1308016.s001.508.pdf]

Environmental Health Perspectives doi:10.1289/ehp.1308016

Supplemental Material

Preconception Maternal and Paternal Exposure to Persistent Organic Pollutants and Birth Size: The LIFE Study

Candace A. Robledo, Edwina Yeung, Pauline Mendola, Rajeshwari Sundaram, Jose Maisog,

Anne M. Sweeney, Dana Boyd Barr, and Germaine M. Buck Louis

Table S1. Geometric means and 95% confidence intervals for untransformed persistent chemical serum concentrations for couples for which a singleton delivery was observed and the standard deviations of the log-transformed persistent chemical serum concentrations used for scaling in analyses, LIFE Study, 2005-2009. 2

Table S2. Adjusted mean changes and their 95% confidence intervals for each birth size measure among girls per 1-SD increase in ln-transformed maternal chemical concentrations for all chemicals evaluated, LIFE Study, 2005-2009. 5

Table S3. Adjusted mean changes and their 95% confidence intervals for each birth size measure among boys per 1-SD increase in ln-transformed maternal chemical concentrations for all chemicals evaluated, LIFE Study, 2005-2009. 8

Table S4. Adjusted mean changes and their 95% confidence intervals for each birth size measure among girls per 1-SD increase in ln-transformed paternal chemical concentrations for all chemicals evaluated, LIFE Study, 2005-2009. 11

Table S5. Adjusted mean changes and their 95% confidence intervals for each birth size measure among boys per 1-SD increase in ln-transformed paternal chemical concentrations for all chemicals evaluated, LIFE Study, 2005-2009. 14

Table S1. Geometric means (GM) and 95% confidence intervals (CI) for untransformed persistent chemical serum concentrations for couples for which a singleton delivery was observed and the standard deviations (SD) of the log-transformed persistent chemical serum concentrations used for scaling in analyses, LIFE Study, 2005-2009.

**Chemical**	**Females GM (95% CI)**	**Males GM (95% CI)**	**Females SD**	**Males SD**
Polybrominated biphenyl (PBB) (ng/g serum)				
153	0.008 (0.007-0.009)	0.012 (0.010, 0.013)	0.0269	0.0487
Organochlorine pesticides (OC) (ng/g serum)				
Hexachlorobenzene	0.046 (0.044, 0.049)	0.054 (0.051, 0.057)	0.0205	0.0234
β-Hexachlorocyclohexane	0.016 (0.014, 0.018)	0.017 (0.015, 0.018)	0.0718	0.0383
γ-Hexachlorocyclohexane (lindane)	0.005 (0.004, 0.006)	0.006 (0.005, 0.007)	0.0020	0.0022
o,p'-DDT	0.002 (0.002, 0.003)	0.003 (0.003, 0.003)	0.0027	0.0025
p,p'-DDT	0.012 (0.011, 0.013)	0.014 (0.013, 0.015)	0.0230	0.0160
Oxychlordane	0.035 (0.032, 0.038)	0.042 (0.039, 0.045)	0.0273	0.0388
trans-Nonachlor	0.051 (0.046, 0.055)	0.068 (0.062, 0.074)	0.0546	0.0844
p,p'-DDE	0.580 (0.534, 0.630)	0.752 (0.700, 0.808)	0.3024	0.2720
Mirex	0.007 (0.007, 0.008)	0.013 (0.011, 0.014)	0.0217	0.0461
Polybrominated diphenyl ethers (PBDE) (ng/g serum)				
17	0.001 (0.001, 0.002)	0.001 (0.001, 0.002)	0.0269	0.0038
28	0.010 (0.009, 0.011)	0.009 (0.008, 0.011)	0.0034	0.0148
47	0.116 (0.101, 0.132)	0.113 (0.099, 0.130)	0.0166	0.2016
66	0.001 (0.001, 0.001)	0.001 (0.001, 0.002)	0.0023	0.0033
85	0.003 (0.002, 0.003)	0.002 (0.002, 0.003)	0.0074	0.0089
99	0.020 (0.018, 0.024)	0.022 (0.019, 0.025)	0.0677	0.0806
100	0.024 (0.021, 0.028)	0.025 (0.021, 0.029)	0.0718	0.0872
153	0.047 (0.041, 0.054)	0.071 (0.006, 0.084)	0.1328	0.2165
154	0.003 (0.002, 0.003)	0.003 (0.002, 0.003)	0.0071	0.0101
183	0.002 (0.001, 0.002)	0.002 (0.002, 0.002)	0.0022	0.0027

Chemical	Females<br>GM (95% CI)	Males<br>GM (95% CI)	Females<br>SD	Males<br>SD
Polychlorinated biphenyls (PCB) (ng/g serum)				
28	0.006 (0.005, 0.007)	0.005 (0.005, 0.006)	0.1201	0.1441
44	0.002 (0.002, 0.002)	0.002 (0.002, 0.002)	0.0155	0.0149
49	0.001 (0.000, 0.001)	0.001 (0.000, 0.001)	0.0060	0.0074
52	0.001 (0.001, 0.001)	0.001 (0.001, 0.001)	0.0151	0.0188
66	0.003 (0.003, 0.003)	0.003 (0.002, 0.003)	0.0324	0.0339
74	0.014 (0.013, 0.015)	0.014 (0.013, 0.015)	0.0280	0.0352
87	0.001 (0.001, 0.002)	0.002 (0.001, 0.002)	0.0013	0.0018
99	0.010 (0.010, 0.011)	0.012 (0.011, 0.013)	0.0091	0.0162
101	0.002 (0.002, 0.002)	0.003 (0.003, 0.003)	0.0043	0.0055
105	0.004 (0.004, 0.004)	0.004 (0.004, 0.004)	0.0035	0.0070
110	0.001 (0.001, 0.002)	0.001 (0.001, 0.002)	0.0020	0.0022
114	0.002 (0.001, 0.002)	0.002 (0.001, 0.002)	0.0014	0.0015
118	0.017 (0.016, 0.018)	0.018 (0.017, 0.019)	0.0139	0.0250
128	0.003 (0.002, 0.004)	0.002 (0.002, 0.003)	0.0008	0.0014
138	0.032 (0.029, 0.034)	0.038 (0.035, 0.042)	0.0285	0.0390
146	0.006 (0.005, 0.006)	0.008 (0.007, 0.008)	0.0055	0.0082
149	0.001 (0.001, 0.002)	0.001 (0.001, 0.002)	0.0046	0.0064
151	0.002 (0.001, 0.002)	0.002 (0.001, 0.003)	0.0058	0.0075
153	0.044 (0.041, 0.048)	0.058 (0.054, 0.063)	0.0386	0.0520
156	0.006 (0.006, 0.007)	0.008 (0.007, 0.008)	0.0060	0.0072
157	0.002 (0.002, 0.002)	0.002 (0.002, 0.003)	0.0018	0.0019
167	0.003 (0.003, 0.004)	0.003 (0.003, 0.003)	0.0021	0.0021
170	0.013 (0.012, 0.014)	0.018 (0.016, 0.019)	0.0114	0.0172
172	0.002 (0.002, 0.003)	0.003 (0.003, 0.004)	0.0019	0.0031
177	0.003 (0.003, 0.003)	0.004 (0.003, 0.004)	0.0052	0.0085
178	0.003 (0.003, 0.004)	0.004 (0.004, 0.005)	0.0032	0.0050
180	0.032 (0.029, 0.034)	0.046 (0.042, 0.05)	0.0276	0.0425
183	0.005 (0.004, 0.005)	0.006 (0.005, 0.006)	0.0063	0.0091
187	0.011 (0.01, 0.012)	0.015 (0.014, 0.017)	0.0140	0.0223

**Chemical**	**Females GM (95% CI)**	**Males GM (95% CI)**	**Females SD**	**Males SD**
189	0.001 (0.001, 0.001)	0.002 (0.001, 0.002)	0.0007	0.0011
194	0.008 (0.007, 0.008)	0.011 (0.010, 0.012)	0.0065	0.0152
195	0.003 (0.002, 0.003)	0.003 (0.003, 0.004)	0.0020	0.0036
196	0.008 (0.007, 0.008)	0.011 (0.010, 0.012)	0.0073	0.0160
201	0.007 (0.007, 0.008)	0.011 (0.009, 0.012)	0.0078	0.0205
206	0.004 (0.004, 0.004)	0.006 (0.005, 0.006)	0.0037	0.0099
209	0.002 (0.002, 0.002)	0.003 (0.003, 0.003)	0.0014	0.0028
Perfluoroalkyl chemicals (PFC) (ng/ml)				
2-(N-ethyl-perfluorooctane sulfonamido) acetate	0.109 (0.102, 0.116)	0.105 (0.100, 0.11)	0.0503	0.0457
2-(N-methyl-perfluorooctane sulfonamido) acetate	0.301 (0.270, 0.336)	0.324 (0.291, 0.361)	0.2460	0.2601
Perfluorodecanoate	0.402 (0.370, 0.437)	0.458 (0.425, 0.494)	0.2100	0.2085
Perfluorononanoate	1.211 (1.127, 1.301)	1.566 (1.462, 1.677)	0.3139	0.3187
Perfluorooctane sulfonamide	0.112 (0.100, 0.125)	0.114 (0.103, 0.125)	0.0351	0.0438
Perfluorooctane sulfonate	12.44 (11.50, 13.44)	21.6 (19.97, 23.39)	0.5506	0.5674
Perfluorooctanoate	3.16 (2.92, 3.42)	5.00 (4.70, 5.32)	0.4348	0.3913

Table S2. Adjusted[a] mean changes (β) and their 95% confidence intervals for each birth size measure among girls per 1-SD increase in ln-transformed maternal chemical concentrations for all chemicals evaluated, LIFE Study, 2005-2009.

Chemical	Birth weight[b] (grams)	Head circumference[c] (cm)	Length[d] (cm)	Ponderal Index[d] (g/cm^3)
PBB 153	18.72 (-64.45, 101.90)	-0.37 (-0.84, 0.11)	0.41 (-0.07, 0.90)	-0.05 (-0.11, 0.01)
HCH	-1.87 (-117.19, 113.46)	0.10 (-0.40, 0.61)	-0.23 (-0.91, 0.46)	0.02 (-0.06, 0.10)
β-HCH	-117.92 (-335.25, 99.42)	-1.47 (-2.33, -0.61)	-0.33 (-1.61, 0.95)	-0.05 (-0.20, 0.11)
γ-HCH (lindane)	1.14 (-93.68, 95.97)	0.03 (-0.37, 0.42)	-0.59 (-1.14, -0.03)	0.09 (0.03, 0.16)
o,p'-DDT	-195.39 (-351.25, -39.52)	-0.78 (-1.48, -0.09)	-0.76 (-1.68, 0.16)	-0.03 (-0.14, 0.08)
p,p'-DDT	-134.91 (-406.39, 136.57)	-1.11 (-2.30, 0.08)	-1.16 (-2.75, 0.43)	0.08 (-0.11, 0.26)
Oxychlordane	-21.48 (-108.40, 65.44)	-0.14 (-0.60, 0.32)	-0.28 (-0.79, 0.23)	0.03 (-0.03, 0.09)
trans-Nonachlor	-5.72 (-85.08, 73.63)	-0.11 (-0.57, 0.34)	-0.20 (-0.66, 0.27)	0.02 (-0.03, 0.08)
p,p'-DDE	4.69 (-140.37, 149.74)	-0.42 (-1.07, 0.24)	-0.12 (-0.97, 0.74)	0.03 (-0.07, 0.13)
Mirex	-68.31 (-275.12, 138.49)	-0.12 (-1.02, 0.78)	0.04 (-1.18, 1.26)	-0.06 (-0.21, 0.08)
PBDE-17	-79.18 (-258.11, 99.75)	-0.50 (-1.41, 0.40)	0.02 (-1.08, 1.13)	-0.06 (-0.21, 0.08)
PBDE-28	-151.33 (-298.56, -4.10)	-1.05 (-1.73, -0.38)	-1.14 (-2.00, -0.28)	0.05 (-0.05, 0.16)
PBDE-47	-98.91 (-269.83, 72.01)	-0.31 (-1.23, 0.61)	-0.91 (-1.90, 0.07)	0.06 (-0.06, 0.18)
PBDE-66	-21.98 (-141.29, 97.33)	-0.17 (-0.76, 0.41)	-0.41 (-1.11, 0.29)	0.05 (-0.04, 0.14)
PBDE-85	14.17 (-186.13, 214.47)	0.34 (-0.78, 1.45)	0.14 (-1.05, 1.34)	-0.02 (-0.17, 0.13)
PBDE-99	52.08 (-120.80, 224.96)	0.27 (-0.48, 1.02)	0.25 (-0.76, 1.27)	-0.01 (-0.13, 0.12)
PBDE-100	-89.66 (-220.08, 40.75)	-0.43 (-1.11, 0.25)	-0.68 (-1.45, 0.08)	0.04 (-0.06, 0.13)
PBDE-153	-22.92 (-114.53, 68.68)	-0.29 (-0.68, 0.11)	-0.25 (-0.78, 0.29)	0.02 (-0.04, 0.09)
PBDE-154	1.51 (-196.27, 199.28)	0.27 (-0.82, 1.36)	0.20 (-0.81, 1.22)	-0.03 (-0.17, 0.11)
PBDE-183	-84.60 (-154.39, -14.82)	-0.27 (-0.58, 0.04)	-0.20 (-0.61, 0.21)	-0.04 (-0.08, 0.01)
PCB-28	-1863.11 (-3746.90, 20.68)	-6.31 (-15.27, 2.65)	-8.82 (-19.95, 2.31)	-0.18 (-1.50, 1.14)
PCB-44	-243.78 (-1395.09, 907.53)	0.82 (-3.84, 5.49)	-2.30 (-7.76, 3.15)	0.15 (-0.59, 0.89)
PCB-49	81.32 (-727.04, 889.67)	1.27 (-2.44, 4.98)	-0.70 (-5.21, 3.81)	0.20 (-0.32, 0.73)
PCB-52	57.54 (-977.11, 1092.20)	2.34 (-2.54, 7.21)	-1.97 (-8.13, 4.19)	0.40 (-0.33, 1.12)
PCB-66	-705.93 (-1815.74, 403.88)	-5.51 (-11.20, 0.17)	-6.05 (-12.57, 0.46)	0.40 (-0.37, 1.17)
PCB-74	-100.48 (-381.23, 180.28)	0.11 (-1.15, 1.37)	-0.35 (-2.01, 1.31)	-0.01 (-0.21, 0.19)

Chemical	Birth weight[b] (grams)	Head circumference[c] (cm)	Length[d] (cm)	Ponderal Index[d] (g/cm^3)
PCB-87	18.55 (-105.13, 142.22)	0.25 (-0.43, 0.93)	-0.34 (-1.06, 0.39)	0.06 (-0.03, 0.14)
PCB-99	-13.74 (-110.24, 82.76)	-0.04 (-0.48, 0.40)	-0.02 (-0.64, 0.61)	0.00 (-0.08, 0.07)
PCB-101	93.69 (-19.89, 207.27)	0.24 (-0.29, 0.76)	0.32 (-0.36, 1.00)	0.02 (-0.06, 0.10)
PCB-105	7.70 (-76.30, 91.71)	0.00 (-0.38, 0.38)	0.05 (-0.44, 0.54)	0.00 (-0.06, 0.06)
PCB-110	64.06 (-37.40, 165.53)	0.17 (-0.29, 0.62)	0.37 (-0.24, 0.97)	-0.01 (-0.08, 0.06)
PCB-114	-6.53 (-88.05, 74.99)	0.02 (-0.34, 0.38)	0.08 (-0.40, 0.56)	-0.01 (-0.07, 0.04)
PCB-118	-5.37 (-94.73, 84.00)	-0.05 (-0.46, 0.36)	-0.09 (-0.61, 0.44)	0.01 (-0.05, 0.07)
PCB-128	13.66 (-68.49, 95.81)	0.08 (-0.30, 0.45)	-0.13 (-0.61, 0.36)	0.03 (-0.03, 0.09)
PCB-138	-82.30 (-219.22, 54.61)	-0.65 (-1.25, -0.05)	0.23 (-0.58, 1.04)	-0.10 (-0.20, -0.01)
PCB-146	-42.22 (-179.65, 95.20)	-0.28 (-0.92, 0.36)	0.10 (-0.76, 0.95)	-0.04 (-0.14, 0.06)
PCB-149	34.35 (-43.22, 111.92)	0.02 (-0.31, 0.36)	0.18 (-0.28, 0.64)	0.00 (-0.06, 0.05)
PCB-151	31.68 (-46.02, 109.37)	0.04 (-0.29, 0.37)	0.20 (-0.26, 0.66)	-0.01 (-0.06, 0.05)
PCB-153	-90.94 (-240.89, 59.01)	-0.65 (-1.30, 0.01)	-0.02 (-0.92, 0.87)	-0.06 (-0.17, 0.04)
PCB-156	-12.26 (-114.71, 90.19)	-0.20 (-0.64, 0.25)	0.29 (-0.31, 0.90)	-0.04 (-0.11, 0.03)
PCB-157	-14.96 (-108.02, 78.09)	-0.22 (-0.62, 0.18)	0.14 (-0.41, 0.70)	-0.03 (-0.09, 0.04)
PCB-167	-61.69 (-172.52, 49.15)	-0.04 (-0.55, 0.46)	-0.47 (-1.12, 0.19)	0.02 (-0.06, 0.10)
PCB-170	-80.87 (-223.93, 62.18)	-0.42 (-1.06, 0.22)	0.28 (-0.57, 1.13)	-0.10 (-0.20, 0.00)
PCB-172	68.59 (-48.81, 185.99)	0.09 (-0.42, 0.61)	0.65 (-0.04, 1.34)	-0.05 (-0.13, 0.03)
PCB-177	50.44 (-44.76, 145.64)	0.03 (-0.37, 0.43)	0.40 (-0.16, 0.96)	-0.03 (-0.09, 0.04)
PCB-178	8.57 (-105.86, 123.01)	-0.18 (-0.67, 0.32)	0.15 (-0.53, 0.83)	-0.02 (-0.10, 0.06)
PCB-180	-36.80 (-180.04, 106.44)	-0.21 (-0.84, 0.42)	0.19 (-0.65, 1.04)	-0.06 (-0.16, 0.05)
PCB-183	25.06 (-79.50, 129.62)	-0.13 (-0.57, 0.31)	0.17 (-0.45, 0.79)	-0.01 (-0.09, 0.06)
PCB-187	49.94 (-62.50, 162.37)	0.09 (-0.39, 0.57)	0.33 (-0.33, 1.00)	-0.02 (-0.10, 0.06)
PCB-189	-12.35 (-117.21, 92.51)	-0.20 (-0.69, 0.30)	-0.20 (-0.82, 0.42)	0.03 (-0.04, 0.11)
PCB-194	44.94 (-70.66, 160.54)	0.26 (-0.24, 0.76)	0.15 (-0.54, 0.83)	0.02 (-0.06, 0.10)
PCB-195	-18.46 (-128.10, 91.18)	0.13 (-0.34, 0.61)	0.03 (-0.62, 0.68)	-0.03 (-0.11, 0.05)
PCB-196	37.27 (-69.43, 143.97)	0.32 (-0.13, 0.77)	0.29 (-0.34, 0.92)	-0.02 (-0.09, 0.06)
PCB-201	72.96 (-28.37, 174.30)	0.51 (0.08, 0.93)	0.19 (-0.41, 0.79)	0.03 (-0.04, 0.10)
PCB-206	71.82 (-26.87, 170.52)	0.52 (0.06, 0.98)	0.21 (-0.38, 0.79)	0.03 (-0.04, 0.10)

Chemical	Birth weight[b] (grams)	Head circumference[c] (cm)	Length[d] (cm)	Ponderal Index[d] (g/cm^3)
PCB-209	-24.96 (-135.66, 85.74)	0.01 (-0.48, 0.51)	-0.26 (-0.92, 0.39)	0.02 (-0.06, 0.10)
Et-PFOSA-AcOH	-43.58 (-148.14, 60.99)	-0.22 (-0.70, 0.26)	0.35 (-0.25, 0.95)	-0.09 (-0.16, -0.02)
Me-PFOSA-AcOH	-13.47 (-107.09, 80.15)	-0.07 (-0.48, 0.34)	0.06 (-0.49, 0.61)	-0.01 (-0.08, 0.05)
PFDeA	-53.42 (-161.01, 54.17)	-0.13 (-0.59, 0.33)	0.02 (-0.61, 0.65)	-0.05 (-0.13, 0.02)
PFNA	-10.08 (-111.46, 91.29)	0.09 (-0.36, 0.54)	-0.22 (-0.83, 0.38)	0.01 (-0.06, 0.08)
PFOSA	-8.80 (-93.55, 75.95)	-0.09 (-0.45, 0.28)	0.34 (-0.16, 0.84)	-0.05 (-0.11, 0.01)
PFOS	14.16 (-81.83, 110.15)	-0.04 (-0.46, 0.38)	0.30 (-0.26, 0.86)	-0.03 (-0.10, 0.03)
PFOA	-61.64 (-159.15, 35.87)	-0.18 (-0.59, 0.23)	-0.17 (-0.74, 0.40)	-0.02 (-0.09, 0.04)

[a]Models are adjusted for maternal and paternal serum lipids, serum cotinine, BMI (kg/m^2), maternal age, difference in parental age, infant gender, the individual and partner sum of remaining chemical concentrations in each chemical's respective class. [b]Data for 113 boys and 117 girls were available for analysis. [c]Data for 90 boys and 91 girls were available for analysis. [d]Data for 113 boys and 116 girls were available for analysis

Table S3. Adjusted[a] mean changes (β) and their 95% confidence intervals for each birth size measure among boys per 1-SD increase in ln-transformed maternal chemical concentrations for all chemicals evaluated, LIFE Study, 2005-2009.

Chemical	Birth weight[b] (grams)	Head circumference[c] (cm)	Length[d] (cm)	Ponderal Index[d] (g/cm^3)
PBB 153	-28.90 (-116.58, 58.78)	0.02 (-0.63, 0.68)	0.04 (-0.47, 0.55)	-0.03 (-0.09, 0.03)
HCH	25.36 (-63.36, 114.07)	0.44 (0.01, 0.87)	0.24 (-0.28, 0.77)	-0.03 (-0.09, 0.03)
β-HCH	22.95 (-58.31, 104.21)	-0.22 (-0.58, 0.14)	0.23 (-0.27, 0.73)	-0.02 (-0.08, 0.05)
γ-HCH (lindane)	54.07 (-31.91, 140.05)	0.33 (-0.03, 0.70)	0.34 (-0.17, 0.84)	-0.01 (-0.07, 0.05)
o,p'-DDT	-6.11 (-93.02, 80.80)	-0.06 (-0.47, 0.35)	0.13 (-0.39, 0.65)	-0.02 (-0.08, 0.04)
p,p'-DDT	3.43 (-85.54, 92.40)	-0.06 (-0.49, 0.36)	0.08 (-0.45, 0.61)	-0.01 (-0.07, 0.05)
Oxychlordane	-3.52 (-99.84, 92.81)	-0.17 (-0.61, 0.26)	0.30 (-0.26, 0.87)	-0.05 (-0.12, 0.01)
trans-Nonachlor	3.92 (-100.89, 108.74)	-0.12 (-0.63, 0.39)	0.21 (-0.41, 0.82)	-0.04 (-0.11, 0.03)
p,p'-DDE	55.59 (-42.16, 153.34)	0.15 (-0.36, 0.65)	0.25 (-0.31, 0.80)	0.03 (-0.04, 0.09)
Mirex	2.15 (-61.46, 65.77)	-0.06 (-0.33, 0.22)	-0.05 (-0.42, 0.33)	0.01 (-0.04, 0.05)
PBDE-17	52.41 (-33.62, 138.44)	0.20 (-0.18, 0.58)	0.31 (-0.21, 0.82)	-0.01 (-0.07, 0.05)
PBDE-28	-64.65 (-164.92, 35.63)	-0.24 (-0.67, 0.19)	-0.18 (-0.76, 0.41)	-0.03 (-0.10, 0.04)
PBDE-47	-12.33 (-168.93, 144.27)	0.30 (-0.53, 1.13)	-0.31 (-1.20, 0.59)	0.03 (-0.09, 0.14)
PBDE-66	125.04 (18.16, 231.92)	0.60 (0.02, 1.18)	0.52 (-0.11, 1.15)	0.02 (-0.06, 0.10)
PBDE-85	113.87 (-46.51, 274.25)	1.04 (0.04, 2.03)	0.63 (-0.35, 1.61)	-0.02 (-0.15, 0.10)
PBDE-99	133.29 (9.21, 257.37)	0.91 (0.23, 1.60)	0.76 (0.04, 1.48)	-0.02 (-0.11, 0.07)
PBDE-100	-39.25 (-199.22, 120.73)	-0.06 (-0.80, 0.67)	-0.50 (-1.43, 0.43)	0.04 (-0.07, 0.16)
PBDE-153	-13.23 (-115.58, 89.11)	-0.15 (-0.60, 0.29)	-0.34 (-0.93, 0.26)	0.05 (-0.02, 0.13)
PBDE-154	93.34 (-91.29, 277.96)	1.01 (-0.34, 2.36)	0.67 (-0.29, 1.63)	-0.03 (-0.16, 0.10)
PBDE-183	85.21 (-32.32, 202.74)	0.25 (-0.47, 0.97)	0.55 (-0.15, 1.25)	-0.02 (-0.11, 0.06)
PCB-28	34.48 (-36.72, 105.68)	0.14 (-0.16, 0.44)	-0.09 (-0.51, 0.33)	0.04 (-0.01, 0.09)
PCB-44	32.46 (-40.00, 104.91)	0.16 (-0.15, 0.47)	-0.10 (-0.53, 0.32)	0.04 (-0.01, 0.09)
PCB-49	34.99 (-37.18, 107.17)	0.16 (-0.15, 0.47)	-0.11 (-0.53, 0.31)	0.05 (0.00, 0.10)
PCB-52	38.39 (-33.58, 110.36)	0.18 (-0.12, 0.48)	-0.10 (-0.53, 0.32)	0.05 (0.00, 0.10)
PCB-66	35.19 (-37.30, 107.67)	0.11 (-0.20, 0.41)	-0.12 (-0.55, 0.30)	0.05 (0.00, 0.10)
PCB-74	34.84 (-41.22, 110.90)	0.17 (-0.15, 0.49)	-0.06 (-0.51, 0.39)	0.04 (-0.01, 0.09)

Chemical	Birth weight[b] (grams)	Head circumference[c] (cm)	Length[d] (cm)	Ponderal Index[d] (g/cm^3)
PCB-87	22.27 (-56.95, 101.49)	0.03 (-0.34, 0.39)	-0.06 (-0.53, 0.40)	0.02 (-0.03, 0.08)
PCB-99	7.49 (-112.41, 127.40)	-0.01 (-0.53, 0.52)	0.01 (-0.71, 0.73)	0.00 (-0.09, 0.09)
PCB-101	104.90 (-2.78, 212.57)	0.20 (-0.29, 0.69)	0.11 (-0.53, 0.75)	0.06 (-0.02, 0.14)
PCB-105	47.30 (-76.53, 171.12)	-0.14 (-0.76, 0.48)	0.35 (-0.36, 1.06)	-0.02 (-0.11, 0.07)
PCB-110	48.93 (-38.88, 136.75)	0.00 (-0.42, 0.42)	0.05 (-0.47, 0.57)	0.03 (-0.04, 0.09)
PCB-114	-29.04 (-154.35, 96.28)	-0.05 (-0.62, 0.51)	0.45 (-0.29, 1.18)	-0.09 (-0.17, 0.00)
PCB-118	-22.98 (-153.65, 107.69)	-0.23 (-0.83, 0.37)	0.01 (-0.76, 0.77)	-0.02 (-0.11, 0.07)
PCB-128	-17.92 (-193.28, 157.43)	-0.86 (-1.67, -0.06)	0.23 (-0.80, 1.26)	-0.05 (-0.18, 0.07)
PCB-138	-149.61 (-285.16, -14.06)	-0.67 (-1.27, -0.08)	0.02 (-0.78, 0.83)	-0.13 (-0.23, -0.04)
PCB-146	-120.50 (-249.74, 8.73)	-0.37 (-1.01, 0.27)	-0.30 (-1.09, 0.50)	-0.05 (-0.14, 0.04)
PCB-149	209.21 (-138.76, 557.18)	-1.22 (-3.31, 0.88)	0.33 (-1.72, 2.39)	0.10 (-0.15, 0.34)
PCB-151	112.45 (-170.76, 395.65)	-1.99 (-5.31, 1.33)	0.36 (-1.31, 2.03)	0.02 (-0.18, 0.22)
PCB-153	-169.93 (-317.32, -22.53)	-0.78 (-1.45, -0.10)	-0.25 (-1.14, 0.64)	-0.10 (-0.20, 0.01)
PCB-156	-81.32 (-194.40, 31.76)	-0.19 (-0.72, 0.34)	-0.02 (-0.70, 0.65)	-0.05 (-0.13, 0.03)
PCB-157	-63.79 (-169.53, 41.94)	-0.17 (-0.66, 0.32)	-0.04 (-0.66, 0.59)	-0.04 (-0.12, 0.03)
PCB-167	-129.24 (-228.16, -30.31)	-0.47 (-0.95, 0.00)	-0.42 (-1.00, 0.16)	-0.03 (-0.10, 0.04)
PCB-170	-153.69 (-288.45, -18.92)	-0.41 (-1.03, 0.21)	-0.10 (-0.90, 0.70)	-0.10 (-0.20, -0.01)
PCB-172	-37.21 (-148.16, 73.74)	-0.19 (-0.70, 0.33)	0.34 (-0.31, 0.99)	-0.09 (-0.17, -0.02)
PCB-177	-10.24 (-202.93, 182.46)	-0.49 (-1.47, 0.50)	0.67 (-0.47, 1.80)	-0.13 (-0.26, 0.01)
PCB-178	-47.14 (-167.33, 73.04)	-0.42 (-0.95, 0.10)	0.07 (-0.65, 0.79)	-0.05 (-0.14, 0.03)
PCB-180	-124.51 (-262.88, 13.85)	-0.31 (-0.93, 0.31)	-0.17 (-0.99, 0.65)	-0.07 (-0.17, 0.02)
PCB-183	-78.43 (-230.70, 73.84)	-0.45 (-1.13, 0.22)	-0.04 (-0.94, 0.86)	-0.07 (-0.18, 0.04)
PCB-187	-50.03 (-188.34, 88.29)	-0.31 (-0.97, 0.35)	0.03 (-0.79, 0.85)	-0.05 (-0.15, 0.04)
PCB-189	-25.09 (-106.28, 56.11)	-0.02 (-0.37, 0.33)	-0.04 (-0.52, 0.44)	-0.02 (-0.07, 0.04)
PCB-194	-26.95 (-134.87, 80.97)	0.09 (-0.39, 0.58)	-0.03 (-0.66, 0.60)	-0.02 (-0.09, 0.06)
PCB-195	-137.73 (-259.57, -15.89)	-0.35 (-0.90, 0.21)	-0.54 (-1.24, 0.16)	-0.05 (-0.14, 0.04)
PCB-196	-50.98 (-180.26, 78.31)	0.05 (-0.58, 0.68)	0.01 (-0.75, 0.77)	-0.05 (-0.14, 0.04)
PCB-201	-5.73 (-125.93, 114.48)	0.28 (-0.31, 0.87)	-0.01 (-0.72, 0.70)	-0.01 (-0.09, 0.08)
PCB-206	5.49 (-90.44, 101.42)	0.16 (-0.30, 0.63)	0.06 (-0.51, 0.62)	0.00 (-0.07, 0.07)

Chemical	Birth weight[b] (grams)	Head circumference[c] (cm)	Length[d] (cm)	Ponderal Index[d] (g/cm^3)
PCB-209	-98.88 (-187.14, -10.61)	-0.21 (-0.59, 0.16)	-0.28 (-0.80, 0.25)	-0.03 (-0.09, 0.03)
Et-PFOSA-AcOH	26.67 (-57.08, 110.43)	-0.21 (-0.62, 0.20)	0.32 (-0.16, 0.80)	-0.02 (-0.08, 0.04)
Me-PFOSA-AcOH	-30.45 (-119.15, 58.25)	-0.07 (-0.45, 0.32)	-0.27 (-0.78, 0.25)	0.03 (-0.03, 0.09)
PFDeA	-1.75 (-90.56, 87.07)	0.04 (-0.34, 0.42)	-0.24 (-0.76, 0.28)	0.03 (-0.03, 0.09)
PFNA	62.66 (-32.05, 157.38)	0.33 (-0.12, 0.77)	-0.17 (-0.74, 0.39)	0.07 (0.00, 0.13)
PFOSA	-104.23 (-194.16, -14.30)	0.00 (-0.42, 0.42)	-0.44 (-0.95, 0.07)	0.02 (-0.04, 0.08)
PFOS	37.51 (-73.45, 148.46)	0.07 (-0.45, 0.60)	0.22 (-0.43, 0.86)	0.00 (-0.07, 0.08)
PFOA	4.78 (-85.44, 95.01)	0.18 (-0.25, 0.60)	-0.24 (-0.77, 0.29)	0.04 (-0.02, 0.10)

[a]Models are adjusted for maternal and paternal serum lipids, serum cotinine, BMI (kg/m^2), maternal age, difference in parental age, infant gender, the individual and partner sum of remaining chemical concentrations in each chemical's respective class. [b]Data for 113 boys and 117 girls were available for analysis. [c]Data for 90 boys and 91 girls were available for analysis. [d]Data for 113 boys and 116 girls were available for analysis.

Table S4. Adjusted[a] mean changes (β) and their 95% confidence intervals for each birth size measure among girls per 1-SD increase in ln-transformed paternal chemical concentrations for all chemicals evaluated, LIFE Study, 2005-2009.

Chemical	Birth weight[b] (grams)	Head circumference[c] (cm)	Length[d] (cm)	Ponderal Index[d] (g/cm^3)
PBB 153	-51.07 (-131.63, 29.50)	-0.10 (-0.42, 0.23)	-0.26 (-0.74, 0.23)	0.00 (-0.06, 0.05)
HCH	-23.54 (-113.46, 66.37)	0.12 (-0.40, 0.63)	0.00 (-0.53, 0.54)	-0.03 (-0.09, 0.04)
β-HCH	54.14 (-142.64, 250.93)	0.23 (-0.68, 1.13)	0.26 (-1.08, 1.61)	0.00 (-0.15, 0.14)
γ-HCH (lindane)	-5.70 (-85.92, 74.52)	0.05 (-0.29, 0.39)	-0.51 (-0.98, -0.04)	0.08 (0.02, 0.13)
o,p'-DDT	-49.28 (-153.83, 55.27)	-0.14 (-0.59, 0.32)	-0.18 (-0.81, 0.44)	-0.02 (-0.09, 0.06)
p,p'-DDT	-18.67 (-157.94, 120.59)	-0.33 (-0.98, 0.32)	0.19 (-0.69, 1.07)	-0.05 (-0.15, 0.05)
Oxychlordane	-36.13 (-138.59, 66.33)	-0.19 (-0.71, 0.32)	-0.07 (-0.69, 0.54)	-0.02 (-0.10, 0.05)
trans-Nonachlor	-24.19 (-119.84, 71.45)	-0.36 (-1.05, 0.32)	-0.31 (-0.88, 0.25)	0.03 (-0.04, 0.10)
p,p'-DDE	37.15 (-100.47, 174.77)	0.09 (-0.56, 0.74)	-0.56 (-1.41, 0.28)	0.12 (0.02, 0.22)
Mirex	-78.47 (-174.27, 17.33)	-0.30 (-0.90, 0.30)	-0.23 (-0.80, 0.33)	-0.04 (-0.10, 0.03)
PBDE-17	-61.41 (-184.28, 61.46)	-0.38 (-0.93, 0.18)	-0.48 (-1.20, 0.24)	0.02 (-0.07, 0.11)
PBDE-28	-30.85 (-154.83, 93.14)	0.02 (-0.57, 0.61)	-0.28 (-1.02, 0.46)	0.01 (-0.08, 0.10)
PBDE-47	-7.26 (-180.19, 165.67)	0.44 (-0.32, 1.21)	-0.51 (-1.55, 0.53)	0.07 (-0.06, 0.19)
PBDE-66	-39.80 (-152.57, 72.96)	0.10 (-0.39, 0.59)	0.06 (-0.63, 0.75)	-0.04 (-0.13, 0.04)
PBDE-85	35.59 (-132.35, 203.53)	-0.01 (-0.83, 0.80)	0.27 (-0.72, 1.26)	-0.03 (-0.15, 0.09)
PBDE-99	17.53 (-123.74, 158.80)	0.10 (-0.52, 0.71)	0.18 (-0.65, 1.01)	-0.02 (-0.12, 0.08)
PBDE-100	44.62 (-99.10, 188.35)	-0.11 (-0.87, 0.64)	0.08 (-0.78, 0.94)	0.03 (-0.07, 0.13)
PBDE-153	10.24 (-65.97, 86.45)	-0.06 (-0.46, 0.34)	0.00 (-0.45, 0.45)	0.01 (-0.04, 0.06)
PBDE-154	-37.34 (-197.47, 122.80)	-0.40 (-1.16, 0.36)	0.08 (-0.86, 1.02)	-0.04 (-0.15, 0.08)
PBDE-183	-92.13 (-173.44, -10.82)	-0.19 (-0.56, 0.18)	-0.42 (-0.93, 0.08)	-0.01 (-0.07, 0.05)
PCB-28	-63.67 (-1069.02, 941.68)	0.96 (-5.23, 7.15)	0.21 (-5.96, 6.37)	-0.13 (-0.92, 0.66)
PCB-44	-192.16 (-919.34, 535.02)	-0.38 (-4.27, 3.51)	-1.67 (-5.40, 2.05)	0.09 (-0.39, 0.57)
PCB-49	-52.11 (-771.62, 667.40)	0.28 (-3.52, 4.08)	-0.55 (-4.55, 3.46)	0.05 (-0.42, 0.53)
PCB-52	-262.98 (-984.22, 458.26)	0.68 (-2.51, 3.87)	-1.19 (-5.52, 3.13)	-0.03 (-0.53, 0.47)
PCB-66	107.13 (-651.54, 865.80)	-0.44 (-4.83, 3.96)	0.68 (-3.86, 5.22)	-0.06 (-0.64, 0.52)
PCB-74	-38.24 (-335.74, 259.27)	-0.24 (-1.59, 1.11)	0.00 (-1.80, 1.81)	-0.06 (-0.28, 0.15)

Chemical	Birth weight[b] (grams)	Head circumference[c] (cm)	Length[d] (cm)	Ponderal Index[d] (g/cm^3)
PCB-87	20.66 (-103.41, 144.74)	-0.21 (-0.86, 0.45)	0.25 (-0.49, 0.99)	-0.03 (-0.12, 0.06)
PCB-99	-43.71 (-131.38, 43.96)	-0.18 (-0.56, 0.20)	-0.24 (-0.82, 0.34)	-0.01 (-0.07, 0.06)
PCB-101	44.45 (-65.63, 154.53)	0.15 (-0.35, 0.65)	0.15 (-0.52, 0.81)	0.01 (-0.06, 0.09)
PCB-105	-39.96 (-121.71, 41.78)	-0.21 (-0.56, 0.14)	-0.19 (-0.68, 0.31)	-0.01 (-0.07, 0.04)
PCB-110	-15.08 (-131.61, 101.46)	-0.05 (-0.69, 0.59)	-0.29 (-1.02, 0.43)	0.04 (-0.04, 0.13)
PCB-114	-14.60 (-98.75, 69.55)	-0.20 (-0.58, 0.18)	-0.11 (-0.63, 0.41)	0.00 (-0.06, 0.06)
PCB-118	-28.78 (-110.84, 53.28)	-0.18 (-0.53, 0.17)	-0.12 (-0.62, 0.38)	-0.01 (-0.07, 0.04)
PCB-128	-21.20 (-120.01, 77.61)	-0.18 (-0.61, 0.25)	0.18 (-0.42, 0.77)	-0.05 (-0.12, 0.02)
PCB-138	-69.04 (-191.78, 53.71)	-0.32 (-0.86, 0.22)	0.15 (-0.57, 0.88)	-0.09 (-0.18, 0.00)
PCB-146	-49.50 (-174.25, 75.25)	-0.23 (-0.80, 0.34)	0.09 (-0.70, 0.88)	-0.06 (-0.16, 0.04)
PCB-149	20.90 (-59.40, 101.20)	0.00 (-0.35, 0.35)	0.18 (-0.32, 0.69)	-0.01 (-0.07, 0.04)
PCB-151	18.87 (-58.61, 96.35)	0.02 (-0.32, 0.36)	0.15 (-0.32, 0.63)	-0.01 (-0.07, 0.05)
PCB-153	-29.33 (-164.37, 105.70)	0.06 (-0.59, 0.71)	0.22 (-0.60, 1.04)	-0.06 (-0.16, 0.03)
PCB-156	-46.65 (-161.20, 67.90)	-0.12 (-0.64, 0.40)	0.29 (-0.39, 0.97)	-0.08 (-0.16, -0.01)
PCB-157	-37.56 (-125.55, 50.43)	-0.06 (-0.46, 0.34)	-0.01 (-0.55, 0.52)	-0.03 (-0.09, 0.04)
PCB-167	-97.49 (-187.45, -7.54)	-0.45 (-0.86, -0.03)	-0.57 (-1.12, -0.02)	0.00 (-0.06, 0.07)
PCB-170	-16.85 (-144.38, 110.67)	0.25 (-0.36, 0.85)	0.09 (-0.69, 0.87)	-0.02 (-0.11, 0.07)
PCB-172	-35.87 (-143.12, 71.38)	-0.02 (-0.50, 0.47)	-0.11 (-0.76, 0.54)	-0.01 (-0.09, 0.07)
PCB-177	15.63 (-74.88, 106.14)	0.07 (-0.32, 0.45)	0.13 (-0.41, 0.68)	-0.01 (-0.07, 0.05)
PCB-178	70.33 (-38.91, 179.57)	0.27 (-0.21, 0.75)	0.36 (-0.31, 1.03)	0.00 (-0.08, 0.08)
PCB-180	25.63 (-104.68, 155.93)	0.54 (-0.09, 1.17)	0.10 (-0.67, 0.87)	0.01 (-0.08, 0.10)
PCB-183	11.91 (-83.74, 107.57)	0.06 (-0.36, 0.48)	0.18 (-0.40, 0.75)	-0.02 (-0.09, 0.05)
PCB-187	41.54 (-64.22, 147.30)	0.22 (-0.23, 0.67)	0.19 (-0.44, 0.82)	0.00 (-0.07, 0.07)
PCB-189	-5.46 (-103.37, 92.45)	0.15 (-0.30, 0.61)	0.16 (-0.42, 0.74)	-0.03 (-0.10, 0.04)
PCB-194	15.68 (-72.06, 103.41)	0.26 (-0.23, 0.75)	-0.11 (-0.63, 0.42)	0.03 (-0.03, 0.10)
PCB-195	-6.94 (-102.96, 89.07)	0.38 (-0.09, 0.85)	-0.20 (-0.77, 0.37)	0.03 (-0.04, 0.10)
PCB-196	4.37 (-81.70, 90.43)	0.23 (-0.21, 0.66)	-0.06 (-0.57, 0.45)	0.02 (-0.04, 0.08)
PCB-201	8.51 (-67.94, 84.96)	0.19 (-0.22, 0.61)	-0.06 (-0.51, 0.39)	0.02 (-0.04, 0.07)
PCB-206	-3.35 (-75.62, 68.92)	0.16 (-0.34, 0.66)	-0.06 (-0.49, 0.37)	0.01 (-0.04, 0.06)

Chemical	Birth weight[b] (grams)	Head circumference[c] (cm)	Length[d] (cm)	Ponderal Index[d] (g/cm^3)
PCB-209	-24.49 (-124.53, 75.56)	-0.04 (-0.84, 0.75)	-0.27 (-0.86, 0.32)	0.02 (-0.05, 0.09)
Et-PFOSA-AcOH	-53.65 (-146.41, 39.11)	-0.09 (-0.49, 0.31)	-0.04 (-0.57, 0.49)	-0.04 (-0.10, 0.03)
Me-PFOSA-AcOH	7.98 (-82.44, 98.39)	0.17 (-0.24, 0.57)	0.11 (-0.43, 0.64)	-0.01 (-0.07, 0.05)
PFDeA	-42.95 (-155.08, 69.17)	-0.16 (-0.65, 0.32)	0.12 (-0.55, 0.79)	-0.06 (-0.14, 0.02)
PFNA	5.15 (-96.09, 106.40)	0.28 (-0.17, 0.72)	-0.19 (-0.79, 0.41)	0.02 (-0.05, 0.09)
PFOSA	10.48 (-85.29, 106.26)	0.07 (-0.34, 0.48)	0.16 (-0.41, 0.72)	-0.01 (-0.07, 0.06)
PFOS	38.58 (-59.29, 136.45)	0.29 (-0.14, 0.71)	-0.05 (-0.62, 0.52)	0.05 (-0.02, 0.11)
PFOA	19.82 (-69.37, 109.02)	-0.03 (-0.42, 0.36)	-0.27 (-0.79, 0.25)	0.06 (0.00, 0.12)

[a]Models are adjusted for maternal and paternal serum lipids, serum cotinine, BMI (kg/m^2), maternal age, difference in parental age, infant gender, the individual and partner sum of remaining chemical concentrations in each chemical's respective class. [b]Data for 113 boys and 117 girls were available for analysis. [c]Data for 90 boys and 91 girls were available for analysis. [d]Data for 113 boys and 116 girls were available for analysis.

Table S5. Adjusted[a] mean changes (β) and their 95% confidence intervals for each birth size measure among boys per 1-SD increase in ln-transformed paternal chemical concentrations for all chemicals evaluated, LIFE Study, 2005-2009.

Chemical	Birth weight[b] (grams)	Head circumference[c] (cm)	Length[d] (cm)	Ponderal Index[d] (g/cm^3)
PBB 153	5.09 (-88.77, 98.95)	-0.22 (-0.63, 0.19)	0.14 (-0.40, 0.69)	-0.02 (-0.09, 0.04)
HCH	2.99 (-118.93, 124.90)	0.18 (-0.40, 0.77)	0.37 (-0.35, 1.09)	-0.06 (-0.14, 0.03)
β-HCH	9.79 (-75.51, 95.09)	-0.16 (-0.78, 0.46)	0.25 (-0.23, 0.74)	-0.03 (-0.09, 0.03)
γ-HCH (lindane)	53.22 (-38.14, 144.58)	0.28 (-0.11, 0.67)	0.33 (-0.20, 0.86)	0.00 (-0.07, 0.06)
o,p'-DDT	4.32 (-86.15, 94.79)	0.20 (-0.28, 0.67)	0.01 (-0.51, 0.54)	-0.01 (-0.07, 0.06)
p,p'-DDT	-22.30 (-126.22, 81.62)	-0.29 (-0.82, 0.25)	0.28 (-0.31, 0.87)	-0.06 (-0.13, 0.01)
Oxychlordane	-85.01 (-197.25, 27.22)	-0.35 (-0.84, 0.14)	-0.19 (-0.85, 0.47)	-0.02 (-0.10, 0.06)
trans-Nonachlor	-71.88 (-176.24, 32.48)	-0.27 (-0.72, 0.19)	-0.37 (-0.98, 0.24)	0.02 (-0.06, 0.09)
p,p'-DDE	16.02 (-81.58, 113.62)	-0.02 (-0.51, 0.46)	0.12 (-0.45, 0.69)	0.01 (-0.06, 0.07)
Mirex	-26.39 (-181.53, 128.75)	-0.12 (-0.84, 0.59)	0.14 (-0.74, 1.01)	-0.06 (-0.17, 0.05)
PBDE-17	34.49 (-95.05, 164.03)	-0.17 (-0.78, 0.43)	0.12 (-0.62, 0.86)	0.00 (-0.09, 0.09)
PBDE-28	14.99 (-99.94, 129.93)	0.18 (-0.33, 0.69)	0.00 (-0.67, 0.67)	0.00 (-0.08, 0.09)
PBDE-47	40.96 (-84.42, 166.34)	0.48 (-0.07, 1.03)	-0.22 (-0.96, 0.52)	0.06 (-0.02, 0.15)
PBDE-66	-47.85 (-173.89, 78.19)	0.33 (-0.26, 0.92)	0.03 (-0.70, 0.77)	-0.04 (-0.13, 0.04)
PBDE-85	95.17 (-89.91, 280.25)	0.16 (-0.76, 1.09)	0.51 (-0.56, 1.58)	-0.02 (-0.15, 0.11)
PBDE-99	59.43 (-90.47, 209.33)	0.31 (-0.38, 0.99)	0.44 (-0.43, 1.31)	-0.03 (-0.13, 0.07)
PBDE-100	54.63 (-58.07, 167.33)	0.00 (-0.51, 0.51)	0.09 (-0.57, 0.75)	0.04 (-0.04, 0.12)
PBDE-153	69.68 (-52.31, 191.66)	0.08 (-0.46, 0.62)	0.29 (-0.40, 0.99)	0.03 (-0.05, 0.12)
PBDE-154	15.79 (-185.14, 216.72)	-0.30 (-1.29, 0.68)	0.25 (-0.92, 1.42)	-0.02 (-0.16, 0.12)
PBDE-183	21.32 (-85.27, 127.91)	0.03 (-0.45, 0.51)	0.30 (-0.32, 0.92)	-0.02 (-0.10, 0.05)
PCB-28	30.99 (-41.13, 103.11)	0.14 (-0.16, 0.44)	-0.12 (-0.54, 0.31)	0.05 (0.00, 0.10)
PCB-44	28.59 (-43.21, 100.39)	0.12 (-0.18, 0.42)	-0.12 (-0.54, 0.30)	0.04 (-0.01, 0.09)
PCB-49	27.27 (-45.01, 99.55)	0.14 (-0.17, 0.45)	-0.13 (-0.56, 0.29)	0.04 (-0.01, 0.10)
PCB-52	25.95 (-46.29, 98.20)	0.12 (-0.19, 0.42)	-0.13 (-0.56, 0.29)	0.04 (-0.01, 0.09)
PCB-66	32.51 (-40.66, 105.68)	0.13 (-0.18, 0.44)	-0.11 (-0.54, 0.32)	0.04 (-0.01, 0.10)
PCB-74	39.33 (-37.79, 116.44)	0.14 (-0.19, 0.47)	-0.03 (-0.48, 0.42)	0.04 (-0.02, 0.09)

Chemical	Birth weight[b] (grams)	Head circumference[c] (cm)	Length[d] (cm)	Ponderal Index[d] (g/cm^3)
PCB-87	37.52 (-53.17, 128.21)	0.01 (-0.52, 0.54)	0.33 (-0.20, 0.87)	-0.03 (-0.09, 0.04)
PCB-99	44.48 (-85.64, 174.59)	0.15 (-0.45, 0.74)	0.02 (-0.75, 0.80)	0.03 (-0.06, 0.13)
PCB-101	57.87 (-58.43, 174.16)	0.20 (-0.40, 0.80)	0.12 (-0.57, 0.81)	0.03 (-0.05, 0.11)
PCB-105	26.19 (-101.10, 153.49)	-0.15 (-1.08, 0.78)	0.35 (-0.40, 1.10)	-0.03 (-0.12, 0.06)
PCB-110	41.50 (-123.29, 40.29)	-0.03 (-0.66, 0.59)	-0.18 (-0.67, 0.30)	-0.01 (-0.06, 0.05)
PCB-114	25.45 (-96.26, 147.16)	-0.14 (-0.74, 0.45)	0.68 (-0.03, 1.39)	-0.09 (-0.17, 0.00)
PCB-118	44.57 (-100.62, 189.77)	0.08 (-0.75, 0.91)	0.43 (-0.42, 1.28)	-0.03 (-0.13, 0.07)
PCB-128	-68.82 (-189.49, 51.85)	-0.66 (-1.31, -0.01)	0.13 (-0.58, 0.84)	-0.07 (-0.16, 0.01)
PCB-138	-103.02 (-264.04, 57.99)	-0.56 (-1.30, 0.17)	0.28 (-0.68, 1.23)	-0.13 (-0.24, -0.02)
PCB-146	-85.33 (-241.25, 70.59)	-0.49 (-1.18, 0.20)	-0.06 (-0.99, 0.87)	-0.06 (-0.17, 0.06)
PCB-149	11.81 (-463.98, 487.61)	-1.94 (-6.04, 2.16)	0.46 (-2.32, 3.24)	-0.08 (-0.41, 0.26)
PCB-151	144.27 (-316.51, 605.06)	-1.04 (-5.25, 3.17)	0.26 (-2.45, 2.97)	0.07 (-0.25, 0.39)
PCB-153	-68.77 (-226.45, 88.91)	-0.24 (-1.02, 0.54)	0.07 (-0.88, 1.01)	-0.06 (-0.18, 0.05)
PCB-156	-74.83 (-198.26, 48.59)	-0.22 (-0.79, 0.36)	0.39 (-0.33, 1.11)	-0.11 (-0.20, -0.03)
PCB-157	-102.66 (-206.35, 1.03)	-0.54 (-1.01, -0.06)	0.07 (-0.55, 0.68)	-0.08 (-0.16, -0.01)
PCB-167	-38.24 (-139.86, 63.37)	-0.32 (-0.80, 0.16)	-0.11 (-0.70, 0.49)	0.00 (-0.07, 0.07)
PCB-170	-119.29 (-268.37, 29.79)	-0.27 (-0.97, 0.43)	-0.38 (-1.26, 0.51)	-0.03 (-0.14, 0.07)
PCB-172	-166.89 (-311.19, -22.60)	-0.63 (-1.28, 0.02)	-0.67 (-1.53, 0.18)	-0.03 (-0.13, 0.07)
PCB-177	-70.92 (-325.22, 183.39)	-0.37 (-1.73, 0.99)	-0.39 (-1.89, 1.11)	0.01 (-0.17, 0.19)
PCB-178	58.16 (-98.32, 214.64)	-0.26 (-0.99, 0.46)	0.13 (-0.80, 1.06)	0.03 (-0.08, 0.14)
PCB-180	-53.71 (-205.47, 98.06)	0.02 (-0.72, 0.76)	-0.36 (-1.25, 0.53)	0.02 (-0.09, 0.12)
PCB-183	-46.98 (-267.32, 173.36)	-0.50 (-1.57, 0.57)	-0.40 (-1.69, 0.88)	0.02 (-0.14, 0.18)
PCB-187	21.30 (-165.91, 208.50)	-0.20 (-1.00, 0.61)	-0.24 (-1.25, 0.78)	0.05 (-0.08, 0.17)
PCB-189	-45.93 (-140.98, 49.12)	-0.27 (-0.70, 0.15)	0.23 (-0.33, 0.79)	-0.06 (-0.13, 0.01)
PCB-194	-45.93 (-187.59, 95.74)	-0.28 (-0.93, 0.37)	-0.40 (-1.23, 0.43)	0.03 (-0.07, 0.13)
PCB-195	-148.39 (-281.69, -15.08)	-0.21 (-0.84, 0.43)	-0.65 (-1.43, 0.13)	-0.02 (-0.11, 0.08)
PCB-196	-52.35 (-217.56, 112.86)	-0.37 (-1.12, 0.39)	-0.50 (-1.47, 0.47)	0.05 (-0.07, 0.16)
PCB-201	-0.68 (-169.89, 168.54)	-0.32 (-1.08, 0.44)	-0.43 (-1.42, 0.56)	0.07 (-0.05, 0.19)
PCB-206	8.76 (-163.09, 180.61)	-0.38 (-1.17, 0.41)	-0.02 (-1.02, 0.98)	0.01 (-0.11, 0.13)

Chemical	Birth weight[b] (grams)	Head circumference[c] (cm)	Length[d] (cm)	Ponderal Index[d] (g/cm^3)
PCB-209	-0.80 (-115.85, 114.24)	-0.35 (-0.88, 0.17)	0.10 (-0.58, 0.78)	-0.01 (-0.09, 0.07)
Et-PFOSA-AcOH	29.10 (-64.65, 122.85)	-0.41 (-1.03, 0.21)	0.51 (-0.05, 1.07)	-0.07 (-0.16, 0.03)
Me-PFOSA-AcOH	-17.27 (-105.41, 70.87)	0.12 (-0.27, 0.51)	-0.20 (-0.71, 0.32)	0.02 (-0.05, 0.08)
PFDeA	23.65 (-64.15, 111.45)	-0.13 (-0.51, 0.26)	-0.14 (-0.65, 0.37)	0.02 (-0.04, 0.09)
PFNA	54.08 (-41.11, 149.28)	0.15 (-0.28, 0.58)	-0.12 (-0.69, 0.44)	0.05 (-0.02, 0.11)
PFOSA	-73.76 (-154.43, 6.91)	0.01 (-0.45, 0.47)	0.01 (-0.46, 0.49)	-0.06 (-0.11, 0.00)
PFOS	36.85 (-73.14, 146.84)	0.16 (-0.37, 0.68)	-0.20 (-0.84, 0.43)	0.06 (-0.02, 0.13)
PFOA	-11.04 (-112.32, 90.23)	-0.04 (-0.52, 0.43)	-0.26 (-0.86, 0.34)	0.03 (-0.04, 0.10)

[a]Models are adjusted for maternal and paternal serum lipids, serum cotinine, BMI (kg/m^2), maternal age, difference in parental age, infant gender, the individual and partner sum of remaining chemical concentrations in each chemical's respective class. [b]Data for 113 boys and 117 girls were available for analysis. [c]Data for 90 boys and 91 girls were available for analysis. [d]Data for 113 boys and 116 girls were available for analysis.
